# Opening the Random Forest Black Box of the Metabolome by the Application of Surrogate Minimal Depth

**DOI:** 10.3390/metabo12010005

**Published:** 2021-12-21

**Authors:** Soeren Wenck, Marina Creydt, Jule Hansen, Florian Gärber, Markus Fischer, Stephan Seifert

**Affiliations:** Institute of Food Chemistry, Hamburg School of Food Science, University of Hamburg, Grindelallee 117, 20146 Hamburg, Germany; soeren.wenck@chemie.uni-hamburg.de (S.W.); marina.creydt@chemie.uni-hamburg.de (M.C.); jule.hansen@chemie.uni-hamburg.de (J.H.); florian.gaerber@studium.uni-hamburg.de (F.G.); markus.fischer@chemie.uni-hamburg.de (M.F.)

**Keywords:** classification, characterization, white asparagus, LC-MS, metabolomics, random forest, feature selection, feature relations, machine learning, chemometrics, surrogate minimal depth

## Abstract

For the untargeted analysis of the metabolome of biological samples with liquid chromatography–mass spectrometry (LC-MS), high-dimensional data sets containing many different metabolites are obtained. Since the utilization of these complex data is challenging, different machine learning approaches have been developed. Those methods are usually applied as black box classification tools, and detailed information about class differences that result from the complex interplay of the metabolites are not obtained. Here, we demonstrate that this information is accessible by the application of random forest (RF) approaches and especially by surrogate minimal depth (SMD) that is applied to metabolomics data for the first time. We show this by the selection of important features and the evaluation of their mutual impact on the multi-level classification of white asparagus regarding provenance and biological identity. SMD enables the identification of multiple features from the same metabolites and reveals meaningful biological relations, proving its high potential for the comprehensive utilization of high-dimensional metabolomics data.

## 1. Introduction

Metabolomics is the in-depth analysis of metabolites, small molecules within biological systems (<1500 Da), that are products of cellular regulatory processes [1]. The composition of the metabolome is directly influenced by environmental factors such as fertilization, soil, climate, or proximity to large bodies of water, with genotype also having a dominant influence [2]. Accordingly, metabolites vary in their chemical structures and concentrations, corresponding to the environmental influences and include, e.g., lipids, sugars, or amino acids. Due to the diversity of compound classes, various technologies and methods have been developed for metabolome analysis, many of which are based on nuclear magnetic resonance (NMR) and mass spectrometry (MS) [3,4]. These techniques are complementary, as NMR technologies probe different substances than MS-based platforms [5]. However, untargeted NMR- and MS data sets are characterized by high numbers of features and small number of samples, which makes the data analysis challenging and prevents the application of classical statistical methods [6].

The popular unsupervised multivariate approach principal component analysis (PCA) reduces the dimensions of data by creating latent variables, which are linear combinations of the original variables. The generated principal components can be used to detect correlations and identify groups with a similar pattern. However, PCA illustrates the main variances in the data set that do not necessarily correspond to the differences that the researcher is interested in [7]. To obtain a model that focuses on these differences, supervised machine learning techniques such as support vector machines (SVM) [8], artificial neural networks (ANN) [9], partial least squares-discriminant analysis (PLS-DA) [10], and random forest (RF) [11] are applied to metabolomics data. RF is particularly suitable because it considers feature interactions and is well suited for high-dimensional data [12].

RF consists of numerous binary decision trees [13]. Each of these decision trees uses a different bootstrap sample containing approximately 63% of the samples (some of them multiple times). For this reason, for each decision tree about 37% of the samples, called out-of-bag (OOB) samples, are left for evaluation, and RF provides independent error estimates (OOB errors) that do not require external data. RF is quite flexible in terms of input and output variables, so it is applied to different data sets, e.g., for classification and regression. To obtain an optimal classification model, the Gini index is applied at each node of each tree to identify the optimal partition among the candidate features. The candidates, whose quantity *mtry* is an important parameter in RF, are randomly selected from all features in each node. However, when RF is applied in a classification setting for prediction, only the assigned class is given and no information about the clarity of the class assignment is provided. In order to close this gap, probability machines have been developed [14]. As their name implies, they generate probabilities that can be used to evaluate the possibility of membership for each class. For this purpose, RF is applied in regression mode providing probabilities for each class that are averaged over all decision trees.

In addition to the application to build classification and regression models, RF can also be used to analyze the importance of individual features and rank them according to their impact on the outcome. This importance measure is either based on the decrease in accuracy of the model when the feature is permuted or the decrease of (Gini) impurity at the nodes that use the respective feature. Since the latter is biased, e.g., in favor of features with many possible split points, an unbiased adaptation called actual impurity reduction (AIR) has recently been introduced [15]. Based on the importance measures, various selection techniques have been developed that separate important from unimportant features. In a comprehensive comparison study, we recently identified Boruta as the best performing approach [16]. Boruta selects features with higher importance than the maximum value of so-called shadow variables, which are obtained by the permutation of the data across observations. For this comparison, a statistical test is applied, assigning significantly larger and smaller importance values. The generation of shadow variables and the comparison is repeated until all variables are labeled or a given number of runs (*maxRuns*) is reached [17].

An alternative approach for feature selection is Surrogate Minimal Depth (SMD), which incorporates relationships into the selection process and does not treat features individually, but as collaborating groups [18]. SMD exploits surrogate variables that have been developed to compensate for missing values in the data [19]. Furthermore, surrogate variables are also used to analyze the relationships between features based on a specific relation parameter that is called mean adjusted agreement. This relation parameter considers the mutual impact of the features to the outcome, and hence goes beyond the analysis of ordinary correlation coefficients. This is why SMD has been successfully applied in various fields and to data sets from different analytical techniques, for example to breast cancer gene expression data [18], FT-NIR food profiling data of hazelnuts [20], and to data from surface-enhanced Raman scattering [21], e.g., for the analysis of drugs in living cells [22].

In this study, we show that RF approaches and, in particular, SMD can be applied to LC-MS data sets for comprehensive multi-level classification and characterization. For this, we use data from asparagus authentication experiments [23,24,25,26,27,28].

## 2. Results and Discussion

### 2.1. Multi-Level Classification

An asparagus LC-MS data set was used for the classification with RF regarding the geographical origin and the botanical variety. In Table 1, the results for the determination of the geographical origin of 213 German, 25 Greek, 31 Dutch, 13 Peruvian, and 35 Polish samples are summarized. An OOB error rate of 12.9% was reached corresponding to a total accuracy of 87.1%. German as well as Greek samples and German as well as Peruvian samples show values above 90% for sensitivity and specificity, respectively, while the Polish samples have values below 70% for both parameters. A detailed evaluation of the performance of the single samples shows that samples from all classes are misclassified as Polish and that the misclassification of German, Dutch, and Polish samples frequently happen among these classes. The reason for these misclassifications probably is the geographical proximity of the North-European samples that were evaluated. Since Germany borders the Netherlands and Poland, less pronounced differences between metabolomes are to be expected here than, for example, in the distinction between Schleswig-Holstein and Bavaria (distance approximately 850 km). Overall, the classification performance regarding the determination of the geographical origin is similar to previous work using LC-MS [26] and other analytical techniques [23,24,25].

The results for botanical diversity classification of 56 Backlim, 23 Cumulus, 42 Gijnlim, and 29 Grolim samples are shown in Table 2. The OOB error and over-all accuracy are 30.0 and 70.0%, respectively, and the values of specificity and sensitivity for the single classes range between 60 and 75%. No misclassification patterns can be identified and the misclassification is evenly distributed among the classes. An exception is the Gijnlim samples, which are frequently misclassified as the variety Backlim. Overall, the classification results are worse than for the determination of the geographical origin. However, previous food profiling techniques that analyze the metabolome of asparagus did not focus on the determination of the variety [23,24,25,26], for which usually the genome is evaluated [29,30]. Hence, with this novel approach, we established a new level for the classification of asparagus LC-MS data showing classification accuracies that are substantially higher than the random distribution of 25%.

The different varieties can be harvested at different times and provide asparagus with different characteristics. For example, the variety Gijnlim provides high yields and rather thin stems right at the beginning of the harvest period, while from the variety Backlim rather thick stems are obtained that can be harvested at the end of the harvest period. For this reason, farmers typically grow several varieties to ensure that there is sufficient product available throughout the harvest season. Samples for this study were all taken in the middle of the harvest season to ensure that, as far as possible, all varieties were present in sufficient numbers. Our results show that the LC-MS metabolome can be utilized to reflect genetic variances caused by different taxonomic varieties of asparagus. However, these are apparently not very large or probably do not affect the composition of the metabolome very much. However, the analysis shows that the metabolome contains comprehensive information that can be used for the detailed RF classification of biological samples on multiple levels.

### 2.2. Probability Machines

**Figure 1:**
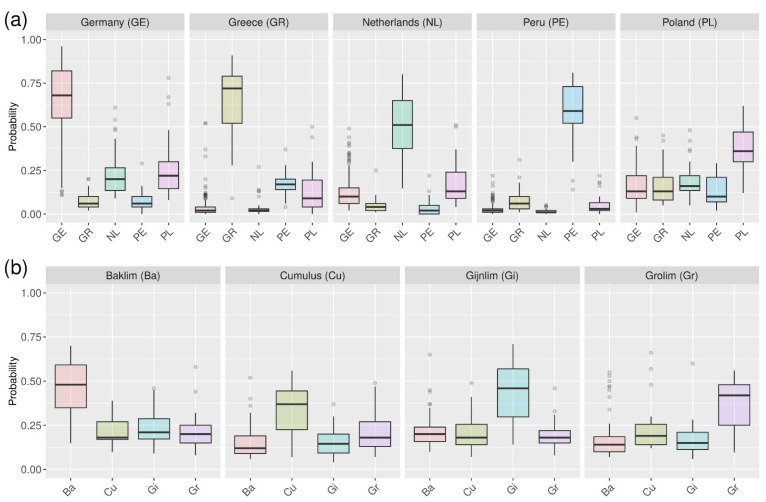
Results of the probability machines for the determination of the geographical origin (**a**) and the botanical variety (**b**). For the samples of each group (title), boxplots are shown that summarize the probabilities for the respective classes.

A disadvantage of classification models regarding their application for unknown samples is that only the final class assignments are reported, and the user does not obtain any information about the clarity of this decision. This is why probability machines have been developed that can be conducted utilizing different machine learning techniques, including RF [14]. For samples that are analyzed by those machines, probabilities for each class are provided that enable a more detailed analysis of the assignment and, if necessary, also the manual labelling as unknown class when the probabilities are too similar. Tables S1 and S2 show the probabilities for each of the samples for the determination of the geographical origin and botanical variety. From these probabilities, boxplots for each class were generated in order to analyze the overall clarity of the decisions. (Figure 1) The assignment for German, Greek, Dutch, and Peruvian samples is quite clear with values mostly above 50% for the respective correct geographical origin (Figure 1a). However, for German and Dutch samples, the probabilities for the respective other North-European samples are slightly increased. This is in agreement with the misclassification patterns that were observed in the confusion matrix in the previous section (see Table 1). It is remarkable that the samples from Poland mainly show comparatively lower probabilities for the correct class assignment of ca. 25% to 50% and the other class assignment have similar probabilities between 10% and 20%. Therefore, it can be concluded that the misclassification of Polish samples is generally caused by a less accurate representation of this group in the classification model and not only by individual samples that come from a region close to the German border.

The probabilities of the correct class assignment for the biological varieties are generally less clear and mainly range between 25% and 60% (Figure 1b). However, they are still higher than the probabilities of the other respective classes that mainly show values between 10% and 30%. Interestingly, the probabilities of the Gijnlim class are slightly higher for the Backlim samples and the probabilities of the Grolim class for the Cumulus class and vice versa. Hence, the LC-MS metabolomes of Backlim and Gijnlim and Cumulus and Grolim are slightly more similar to each other, which was not apparent from the confusion matrix in the previous section (see Table 2). This demonstrates that the analysis of probabilities can give valuable additional insights about the similarities of the analyzed classes.

### 2.3. Feature Selection

**Figure 2:**
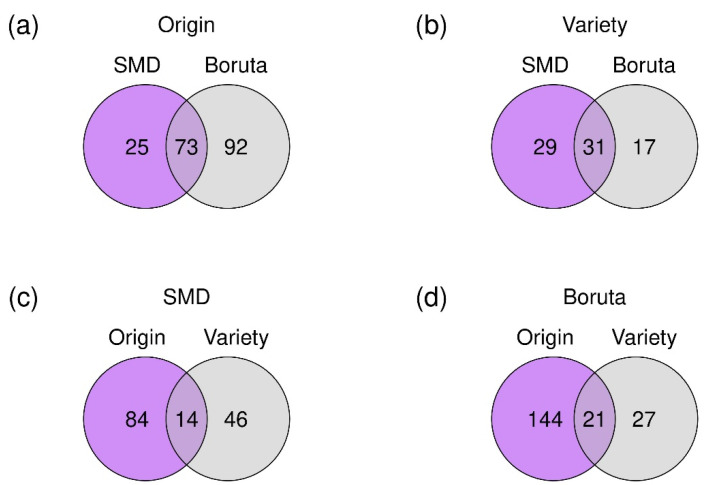
Venn diagrams of selected features for the classification on the different levels of geographical origin (**a**) and botanical variety (**b**) utilizing the approaches SMD (**c**) and Boruta (**d**). Detailed lists of the selected features can be found in Tables S3 and S4.

In order to characterize the differences between the classes of asparagus samples, we applied the feature selection approaches SMD and Boruta. For the determination of the geographical origin 98 features with SMD and 165 features with Boruta and for the botanical variety 60 features with SMD and 48 features with Boruta were selected. Lists of the selected features are given in Tables S3 and S4. The overlap of the different approaches and the classification levels is visualized separately in Figure 2 and together in Figure S1. The Venn diagrams show that many features are selected by both approaches (Figure 2a,b). The differences are caused by the fact that the mutual importance of multiple metabolites is evaluated by SMD, while Boruta evaluates the metabolites individually. The small overlap between different classification levels that are depicted in Figure 2c,d show that mainly different metabolites are responsible for the classification regarding provenance and botanical variety. This means that mostly different subsets of the metabolites are utilized to build the different classification models.

### 2.4. Analysis of the Relations of Selected Features

**Figure 3:**
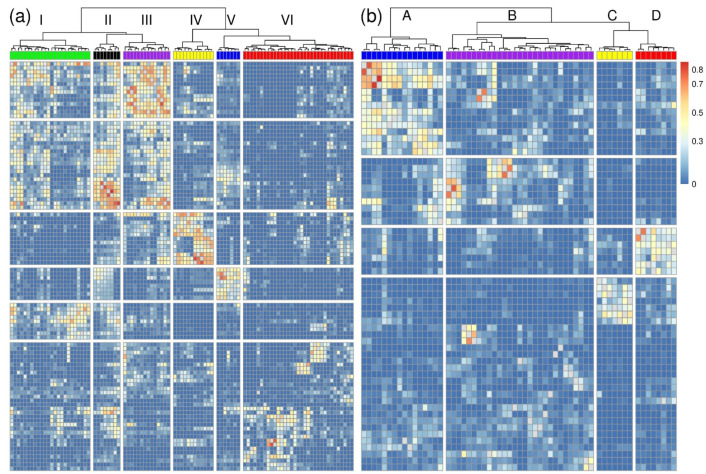
Results of the relation analysis of selected features with SMD for the determination of geographical origin (**a**) and botanical variety (**b**). For the hierarchical cluster analyses, Euclidean distances and Ward algorithm were applied and the clusters are labeled with I–VI and A–D. The clusters were assigned to the following molecular groups: I: Triaclyglycerols, II: Oxylipins, III: acylated monogalactosyldiacylglycerols, IV: Phytosterol esters, V: Triacylglycerols, VI: Various, A: Cycloartenol derivates, B: Coenzymes Q9 and Q10, C: Stigmasterol derivates, D: Diacylglycerols.

We applied SMD in order to analyze the relations between the selected features. As a result, for each pairwise feature combination, the relation parameter mean adjusted agreement is obtained, which represents the mutual impact on the outcome (see Tables S5 and S6). For a comprehensive characterization of the class differences, we depicted the results in a heatmap that was obtained by the application of cluster analysis. We generated heatmaps for both, the selected features by Boruta and SMD. The comparison of the heatmaps of the different approaches confirms the assumption from the previous section that Boruta mainly selected important individual features, while SMD selected important groups (compare Figure 3 with Figures S2 and S3).

**Figure 4:**
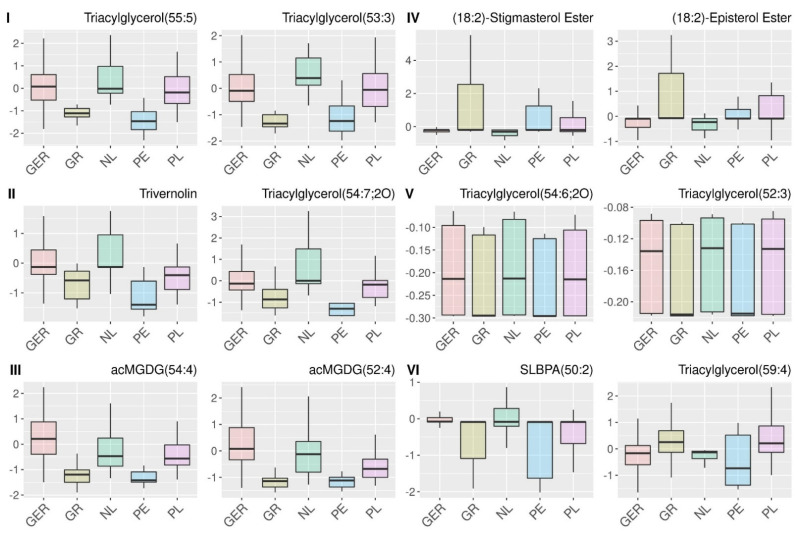
Boxplots of the autoscaled intensities for two exemplary metabolites of the clusters (**I**–**VI**) from the relation analysis Figure 3. A. Detailed information about the metabolites can be obtained from Table S7. Abbreviations: acMGDG: acylated monogalactosyldiacylglycerols; SLBPA: semi-lyso-bis-phosphatidic acid.

Figure 3a shows the results for the 98 features selected by SMD for the determination of the geographical origin. Six distinct clusters I-VI that contain metabolites with mainly similar information for the classification are obtained. In Figure 4, the intensities of two representative metabolites are shown for each cluster, while boxplots for all selected features are depicted in Figures S4–S9. The clusters I–III contain metabolites with similar classification patterns to separate North-European samples with higher intensities from the Peruvian and Greek samples that show lower intensities. Each of these clusters are mainly associated with one specific molecular class. Cluster I contains triacylglycerols with mono or double unsaturated fatty acids (Figure 4I). The degree of saturated and unsaturated fatty acids of plants is related to biotic and abiotic stress induced by external influences, which is why fatty acids have already been utilized to distinguish different geographical origins of food [26,31,32,33]. Cluster II consists of triacylglycerols with epoxy-fatty acids like trivernolin (Figure 4II). Cluster III contains different acylated monogalactosyldiacylglycerols (acMGDG) that also have previously been related to biotic and abiotic stress [32,34,35,36] and were identified as markers for the identification of German asparagus samples [26,37]. (Figure 4III) The high relations between the clusters I–III can be explained by the fact that all, triacylglycerols, triacylglycerols with epoxy-fatty acids, and MGDGs, are involved in the lipid metabolism of plants to build oxylipins as an environmental response [36]. Oxylipins are major actors in plant defense and have been shown to counteract bacterial and fungal infestation of plants [36,38]. Hence, it is very plausible that these metabolites in those clusters are useful interacting markers for the determination of the geographical origin of asparagus.

Arguably the most interesting cluster for interpretation is cluster IV, which can be applied to distinguish Dutch and German from the other samples that have higher intensities. This cluster contains several (18:2)-Phytosterol esters, e.g., (18:2)-Stigmasterol ester and (18:2)-Episterol ester, but also (18:2)-Campesterol ester and (18:2)-Sitosterol ester. (Figure 4IV). Also, the molecules of this class that interact in the sterol biosynthesis pathway have previously been identified as important for the determination of German asparagus samples because those compounds are affected by environmental changes [26,39,40].

Cluster V shows a similar but slightly different grouping of the classes as the clusters I to III and consists of triacylglycerols with multiple double bonds and their epoxides (Figure 4V). However, since those metabolites are arranged in a separate cluster, it can be concluded that they contribute to the classification model in essentially other ways. Cluster VI is quite diverse, including various metabolites such as triacylglycerols and phospholipids like semi-lyso-bis-phosphatidic acids (SLBPAs), which contribute differently to the separation of the geographical origins. (Figure 4VI).

**Figure 5:**
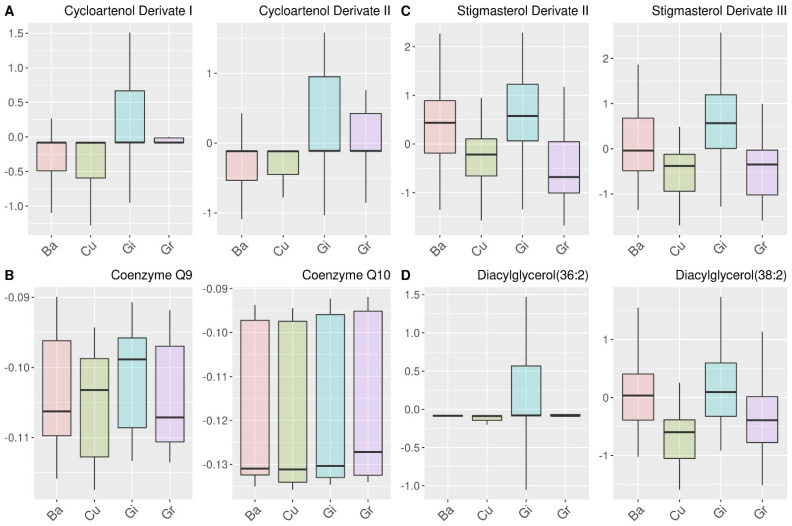
Boxplots of the autoscaled intensities for two exemplary features of the clusters (**A**–**D**) from the relation analysis for the determination of botanical variety in Figure 3B. Detailed information about the metabolites can be obtained from Table S7.

Similar as for the classification of the geographical origin, the selected features for the determination of the botanical variety were evaluated. Figure 3b shows the results of the relation analysis, while Figure 5 shows boxplots of two representative features for each cluster, and all features are depicted in Figures S10–S13. Also, here the clusters mainly contain metabolites that carry similar information for the classification. Cluster A is characterized by cycloartenol derivates that differentiate between low intensities of the varieties Backlim and Cumulus and high intensities of Gijnlim and Grolim (Figure 5A). Cycloartenol has been identified as the precursor for phytosterols influencing membrane fluidity, which is relevant in colder regions [41]. Cluster B consists of different metabolites that also contain various information for the variety classification. (Figure 5B) These metabolites include coenzymes Q9 and Q10, both of which are ubiquinones and act as carriers of electrons in mitochondrial membranes [42] and are involved in the abiotic stress response of plants [43]. Cluster C and D contain stigmasterol derivates and diacylglycerols and both show higher intensities for the varieties Backlim and Gijnlim. Hence, the metabolites in these clusters are responsible for the similarities of those botanical varieties that we observed by the analysis of class similarities by probability machines in Section 3.2.

To summarize: The relation analysis that is accessible by the application of SMD enables the comprehensive characterization of the LC-MS metabolome of biological samples. This analysis goes beyond the analysis of pairwise correlation coefficients, which is demonstrated by the comparison of the SMD relation analysis results (Figure 3) to heatmaps that were generated based on pairwise Pearson correlation coefficients (Figures S14 and S15). The correlation coefficients do not show the biological context of the metabolites that can be revealed by SMD, which is apparent by the clear grouping of molecule classes, e.g., of the phytosterol esters (Figure 3a, cluster IV) and the Coenzymes (Figure 3b, cluster B). In future applications, metabolites with mutual impact identified by SMD could be simultaneously utilized to directly test for specific characteristics, e.g., by the application of pathways-guided random forest approaches [44].

**Figure 6:**
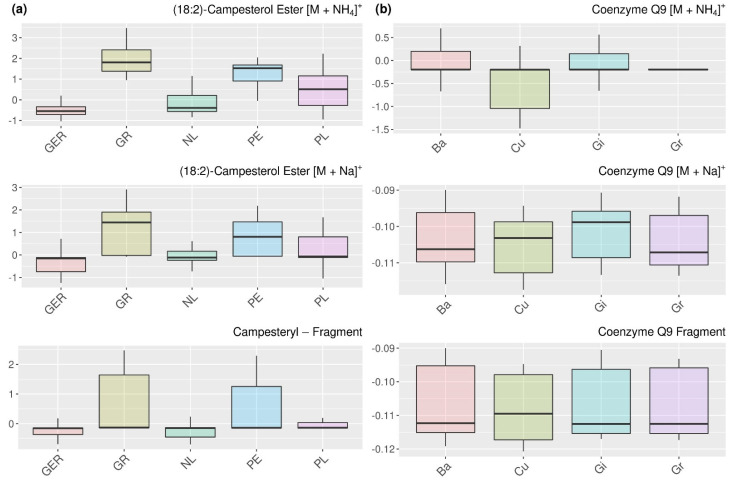
Comparison of features with very high pairwise mean adjusted agreement values that could be assigned to the same metabolites. Features of campesterol ester for the relation analysis regarding the determination of geographical origin (**a**) and features of coenzyme Q9 for the botanical variety (**b**) are shown. Detailed information about the metabolites can be obtained from Table S7.

In all the clusters that we comprehensively analyzed in this section, small groups of highly related features could be identified. Those groups that consist of two to three elements contain very similar information for the classification, and we could assign those features to the same metabolites. In Figure 6, this is exemplarily shown for campesterol ester (a) and coenzyme Q9 (b). Since we always retained the [M − NH_4_]^+^ adduct in preprocessing, it is not surprising that the respective features can be identified in addition to the [M + Na]^+^ adduct that is commonly detected. In addition, we also found typical molecular fragments formed from these molecules during the mass spectrometric measurement, which could be merged based on their mean adjusted agreement. Hence, SMD relation analysis in combination with an appropriate threshold could be applied in addition to correlation coefficients in LC-MS data processing workflows in order to merge features from the same metabolites.

## 3. Materials and Methods

### 3.1. Data Acquisition and Preprocessing

An LC-MS data set containing 317 samples obtained in the years 2016, 2017, and 2018 was used. For detailed information about the LC-MS measurement, please refer to [37]. The raw data set comprise the following positively charged adducts: [M + Na]^+^; [M + K]^+^; [M − H_2_O + H]^+^; [M − CO_2_ + H]^+^; [M − NH_3_ − H]^+^. In order to reduce the features from the same compounds, only the one with the highest intensity was used. Potential ammonium adducts were taken into account separately, since in a previous study, in some cases incorrect binning was observed [37]. Subsequently, the data set was further processed by excluding features that are present in less than 80% of the samples resulting in 718 features. In addition, missing data points were imputed with missForest [45] and, since the samples were measured in three batches, autoscaling was conducted separately for each batch [46]. In Figure S16, it can be observed that autoscaling significantly reduced the batch effects. For the identification of the important metabolites, MS/MS experiments were carried out for the features that were selected by SMD (see Table S7).

For the determination of the geographical origin, all 317 samples assigned to the five classes, Germany, Greece, Netherlands, Peru, and Poland were used (Table 3). In addition, some samples were used to differentiate between varieties. The focus was on the four most commonly grown varieties in Germany: Backlim, Cumulus, Gijnlim, and Grolim (Table 4).

### 3.2. Software and Analyses

The software R (version 3.6.3) and the R packages missForest (version 1.4, CRAN) [45] for missing value imputation, Pomona (Version 1.0.1, https://github.com/silkeszy/Pomona, accessed on 20 December 2021) [16] for Boruta feature selection, SurrogateMinimalDepth (version 0.2.0, https://github.com/StephanSeifert/Surrogate MinimalDepth, accessed on 20 December 2021) [18] for SMD feature selection and relation analysis, ranger (version 0.12.1, CRAN) [47] for RF analysis (classification and probability forest) and mdatools (version 0.12.0, CRAN) for PCA were used [48].

The RF approaches were applied in classification and probability mode with the parameters summarized in Table 5. In order to compensate for the class imbalance, the parameter *case.weights* was chosen accordingly. This means that the samples from rare classes were sampled more frequently for the bootstrap samples to train the RF. For visualization of the results of variable relation analysis, heatmaps of the mean adjusted agreement values of important features were depicted by the R package pheatmap using hierarchical cluster analysis with Euclidean distance and Ward algorithm [49]. For comparison, heatmaps of the Pearson correlation coefficients were generated accordingly.

## 4. Conclusions

In this study, we demonstrate the enormous potential that RF approaches, and SMD in particular, provide for the extensive exploitation of high-dimensional LC-MS data. We do this through their application to the data of biological samples, which goes far beyond black box classification. To be more precise, the classification of an asparagus data set regarding provenance and botanical varieties is complemented by the detailed evaluation of the class similarities obtained by the application of RF probability machines and the characterization by feature selection and relation analysis. For the relation analysis, we investigate the mutual impact of the features on the outcome that is accessible by SMD. This approach is very promising to get a comprehensive picture of the complex impact of metabolites on the outcome, as it reveals specific molecular groups and biomolecules known to interact in biological pathways.

## Figures and Tables

**Table 1:** Confusion matrix for the classification of the geographical origin.

	Germany	Greece	Netherlands	Peru	Poland	Sensitivity [%]
Germany	196	4	5	0	8	92.0
Greece	0	23	0	0	2	92.0
Netherlands	5	0	23	0	3	74.2
Peru	1	0	0	11	1	84.6
Poland	7	2	3	0	23	65.7
Specificity [%]	93.8	79.3	74.2	100	62.2	

**Table 2:** Confusion matrix for the classification of the botanical variety.

	Backlim	Cumulus	Gijnlim	Grolim	Sensitivity [%]
Backlim	41	2	6	7	73.2
Cumulus	3	14	3	3	60.9
Gijnlim	9	1	31	1	73.8
Grolim	1	4	3	19	65.5
Specificity [%]	73.2	66.7	72.1	63.3	

**Table 3:** Overview of the analyzed samples regarding geographical origin.

Origin	2016	2017	2018
Germany	105	77	31
Greece	14	7	4
Netherlands	10	10	11
Peru	7	4	2
Poland	16	11	8

**Table 4:** Overview of the analyzed samples regarding botanical variety.

Variety	2016	2017
Backlim	33	23
Cumulus	12	11
Gijnlim	22	20
Grolim	18	11

**Table 5:** Parameters used for the RF-based approaches with p representing the total number of features.

Approach	Parameter	Description	Value
RF	ntree	number of trees	10,000
	min.node.size	number of samples in terminal node	1
	mtry	number of candidate features	138 (p^3/4^) ^1^
	case.weights	weights for sampling of training observations	chosen according to the size of the respective class
Boruta	importance	applied importance measure	impurity_corrected
	pValue	confidence level	0.01
	maxRuns	maximum number of importance source runs	100
SMD	*s*	predefined number of surrogate splits	35 (p ∙ 0.05)

^1^ Motivated by [50].

## Data Availability

The unnormalized feature table is included in the Supplementary Materials.

## Supplementary Materials

The following are available online at www.mdpi.com/2218-1989/12/1/5/s1, Figure S1: Venn diagram of all selected features, Figures S2 and S3: Results of the relation analysis of features selected by Boruta for the determination of geographical origin (S1) and botanical variety (S2), Figures S4–S9: Boxplots of the autoscaled intensities for the features of the cluster I-VI from the relation analysis for the determination of geographical origin in Figure 3a, Figures S10–S13: Boxplots of the autoscaled intensities for the features of the clusters A–D from the relation analysis for the determination of botanical variety in Figure 3b, Figures S14 and S15: Results of the correlation analysis of features selected by SMD for the determination of geographical origin (S14) and botanical variety (S15), Figure S16: Results of the PCA showing the sample distribution before and after autoscaling, Tables S1 and S2: Results of the probability machines for the determination of geographical origin (S1) and botanical variety (S2), Tables S3 and S4: Results of variable selection for the determination of geographical origin (S3) and botanical variety (S4), Tables S5 and S6: Results of variable relation analysis for the determination of geographical origin (S5) and botanical variety (S6), Table S7: Identified key metabolites for the separation of asparagus samples, Table S8: Feature table.
