# Supplementary material for: Opening the Random Forest Black Box of the Metabolome by the Application of Surrogate Minimal Depth
Source: Metabolites. 2021 Dec 21;12(1):5. doi: 10.3390/metabo12010005 (PMC8781913; doi:10.3390/metabo12010005)
Supplement: Supplementary file 1 [file metabolites-12-00005-s001.zip › metabolites-1490989-supplementary/Supplementary Figures.pdf]

Supplementary Figures

Opening the random forest black box of the metabolome by the application of surrogate minimal depth

Soeren Wenck, Marina Creydt, Jule Hansen, Florian Gärber, Markus Fischer and Stephan Seifert*

Hamburg School of Food Science – Institute of Food Chemistry, University of Hamburg, Grindelallee 117, 20146 Hamburg, Germany

*Corresponding Author
E-Mail: stephan.seifert@chemie.uni-hamburg.de; Phone: +49 40 428 38 8818

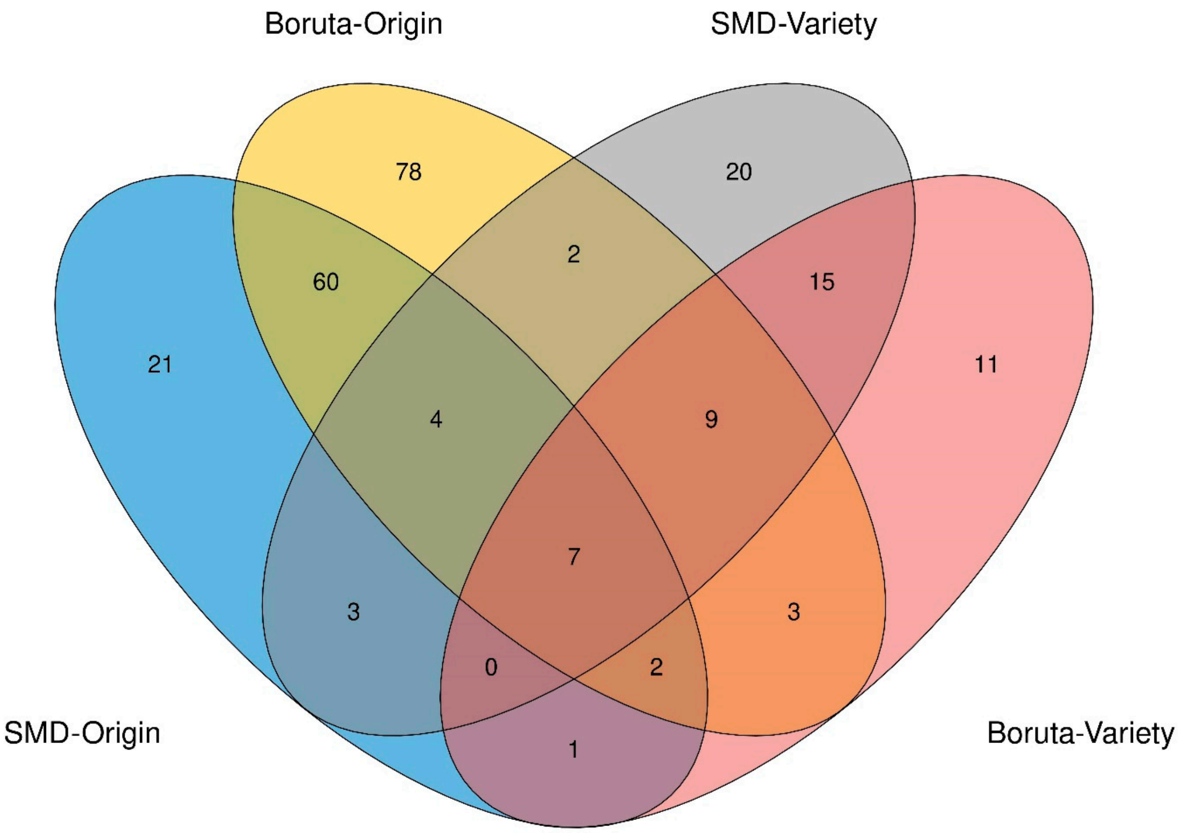

Figure S1 Visualization of selected features for the classification on the different levels of geographical origin and botanical variety utilizing the approaches SMD and Boruta shown in one Venn diagram. Detailed lists of the selected features can be found in Table S3 and S4.

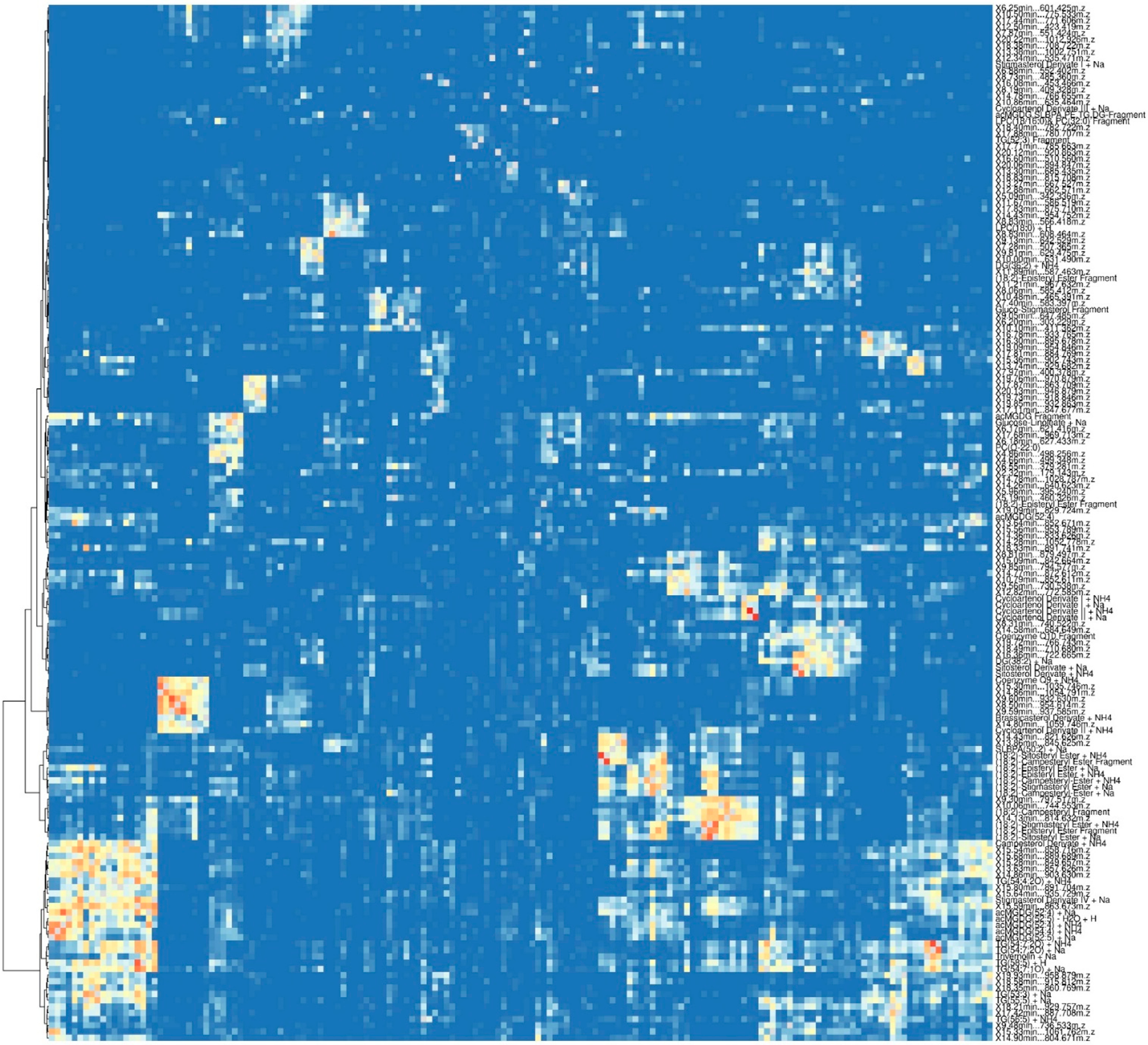

Figure S*2*. Results of the relation analysis of features that were selected by Boruta for the determination of geographical origin. For the hierarchical cluster analyses Euclidean distances and Ward algorithm were applied.

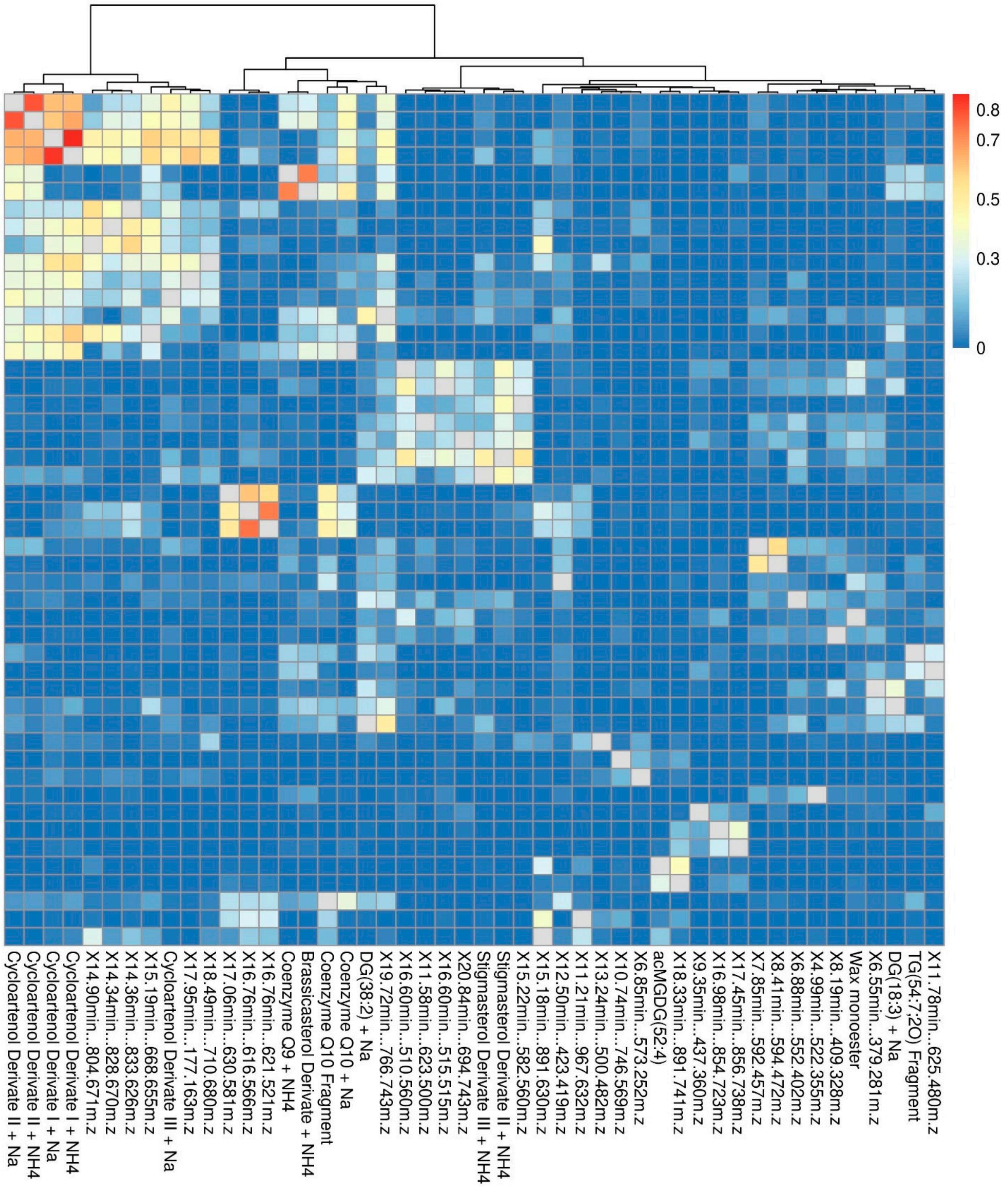

Figure S3. Results of the relation analysis of features that were selected by Boruta for the determination of botanical variety. For the hierarchical cluster analyses Euclidean distances and Ward algorithm were applied.

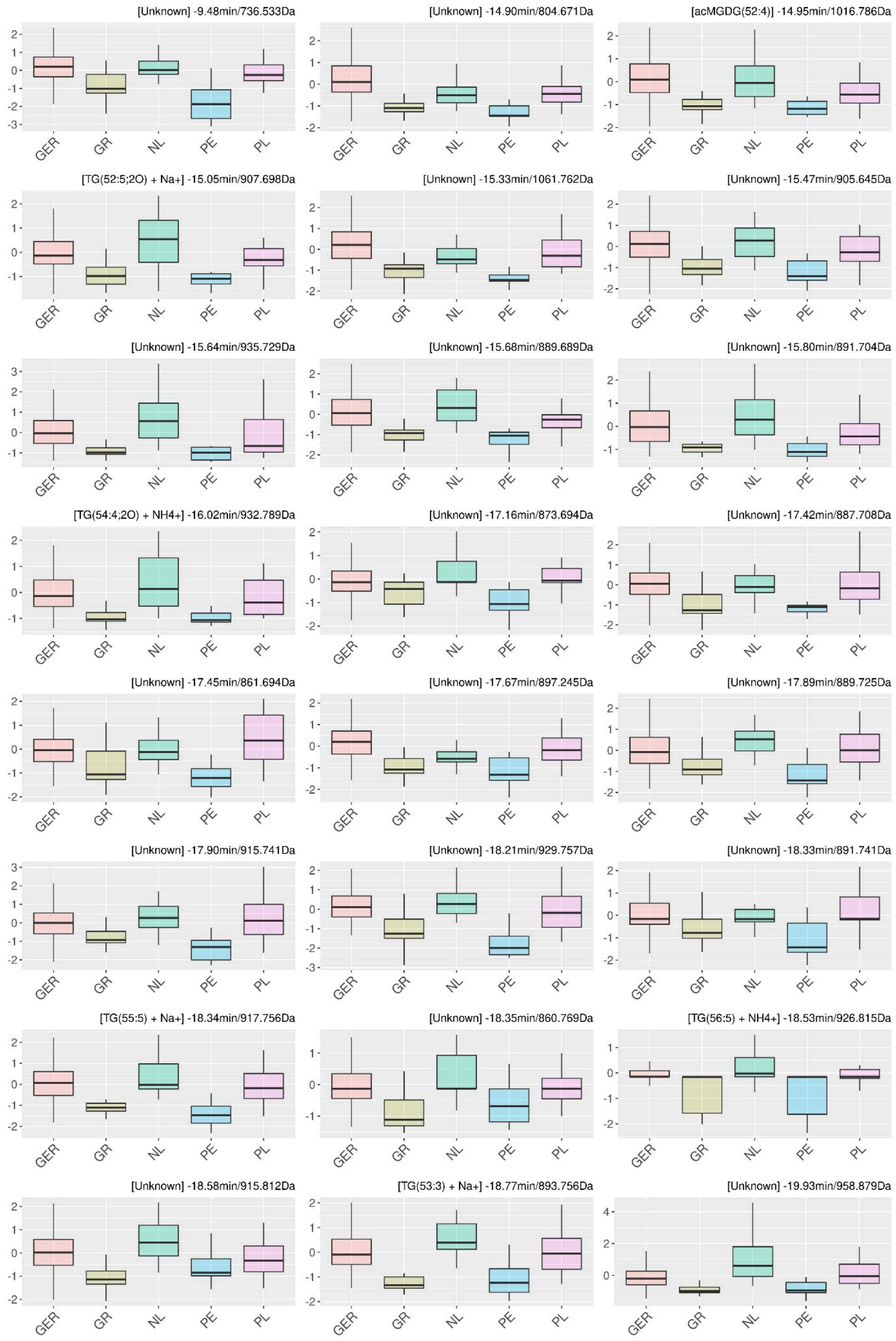

Figure S4. Boxplots of the autoscaled intensities for the features of the cluster I from the relation analysis for the determination of geographical origin in Figure 3a. Detailed information about the metabolites can be obtained from Table S3.

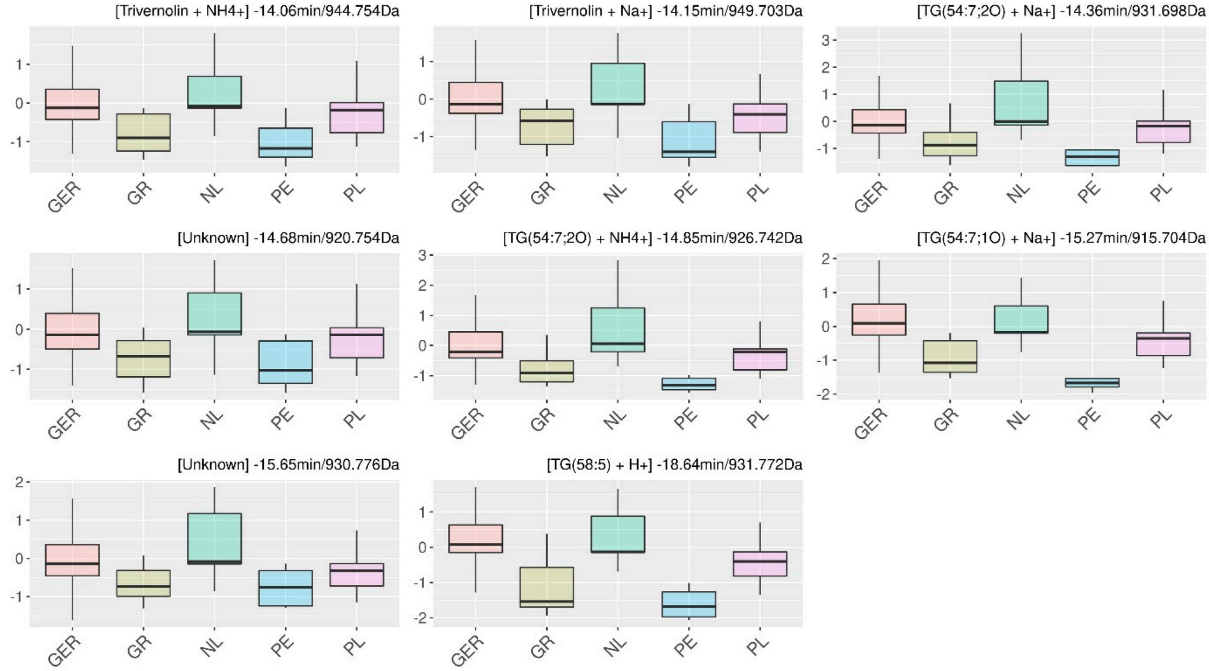

Figure S5. Boxplots of the autoscaled intensities for the features of the cluster II from the relation analysis for the determination of geographical origin in Figure 3a. Detailed information about the metabolites can be obtained from Table S3.

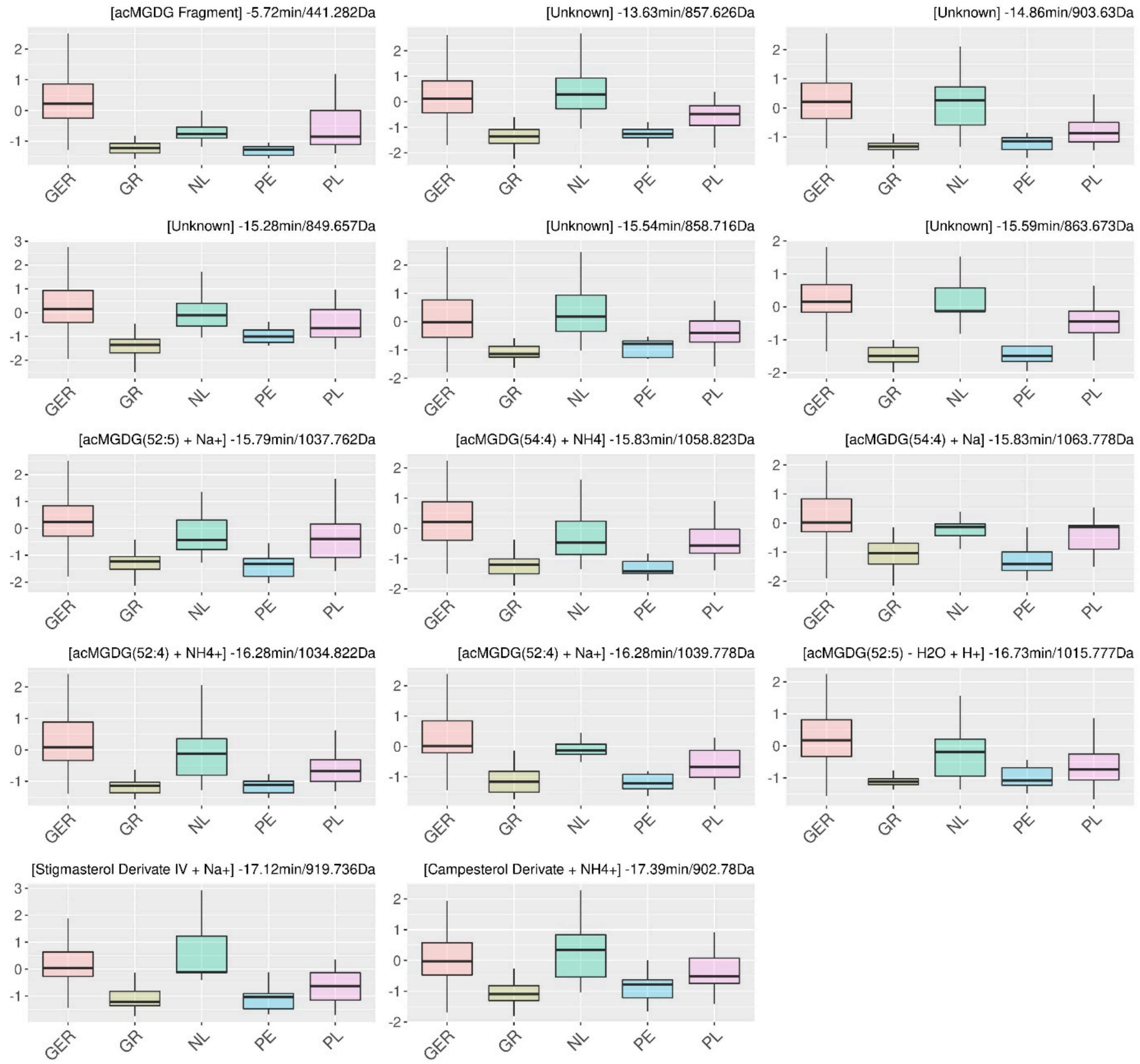

Figure S6. Boxplots of the autoscaled intensities for the features of the cluster III from the relation analysis for the determination of geographical origin in Figure 3a. Detailed information about the metabolites can be obtained from Table S3.

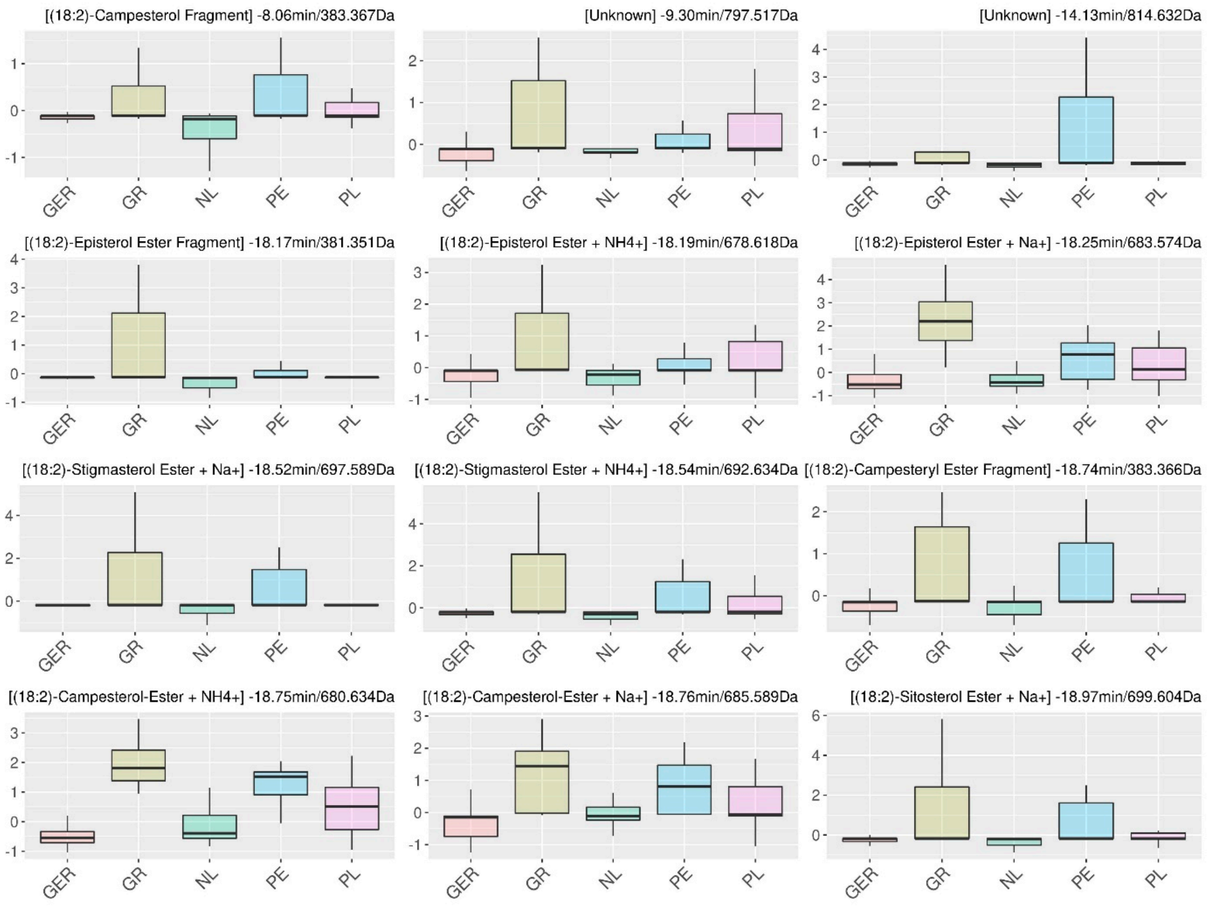

Figure S7. Boxplots of the autoscaled intensities for the features of the cluster IV from the relation analysis for the determination of geographical origin in Figure 3a. Detailed information about the metabolites can be obtained from Table S3.

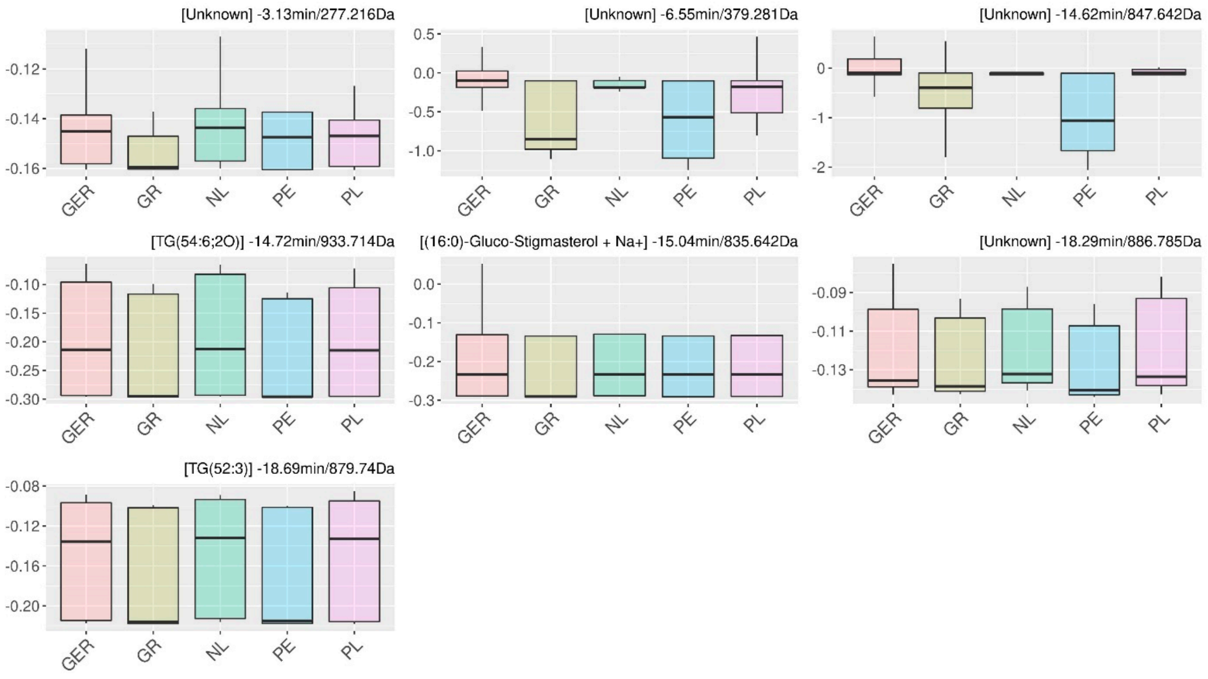

Figure S8. Boxplots of the autoscaled intensities for the features of the cluster V from the relation analysis for the determination of geographical origin in Figure 3a. Detailed information about the metabolites can be obtained from Table S3.

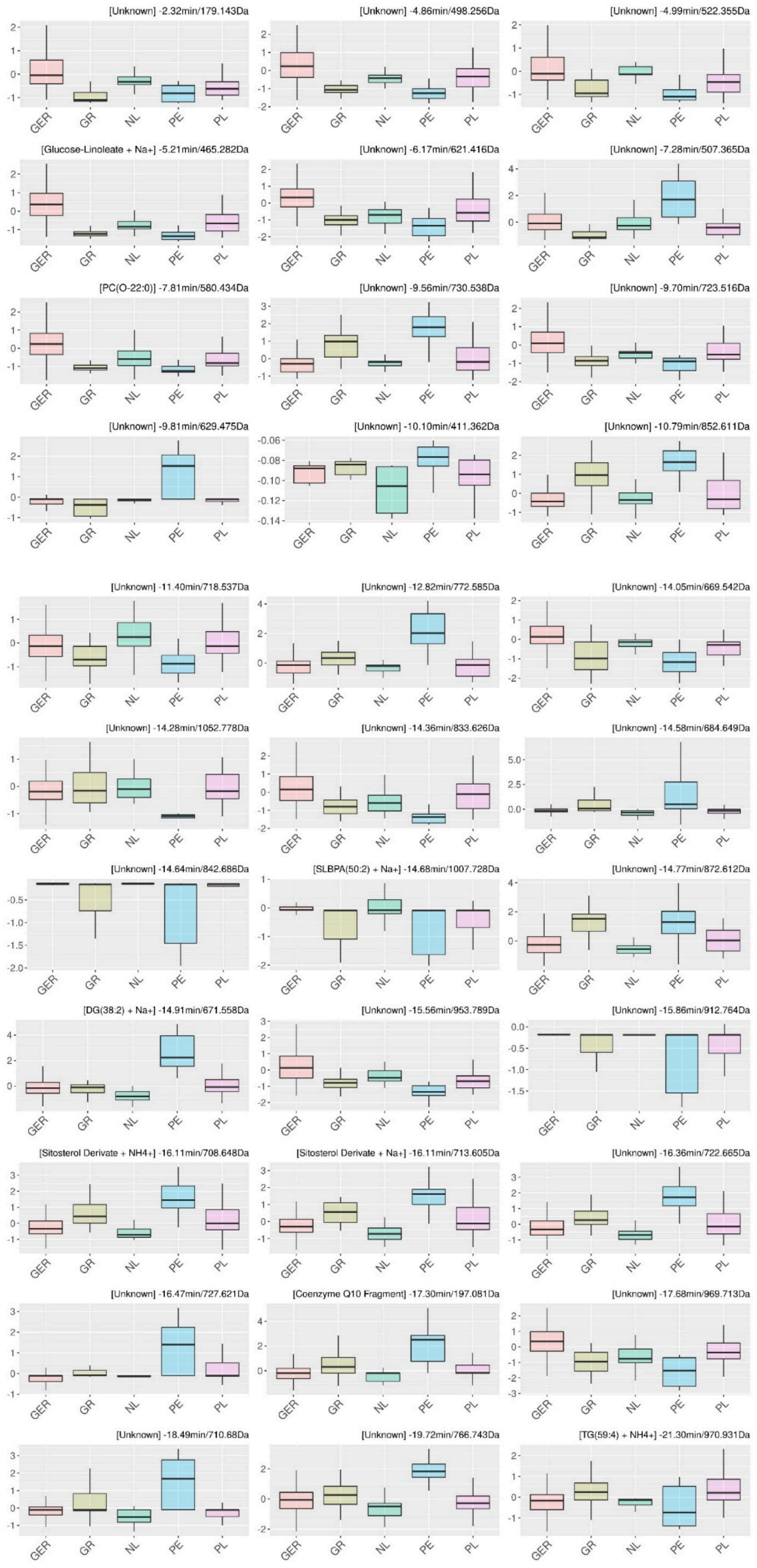

Figure S9. Boxplots of the autoscaled intensities for the features of the cluster VI from the relation analysis for the determination of geographical origin in Figure 3a. Detailed information about the metabolites can be obtained from Table S3.

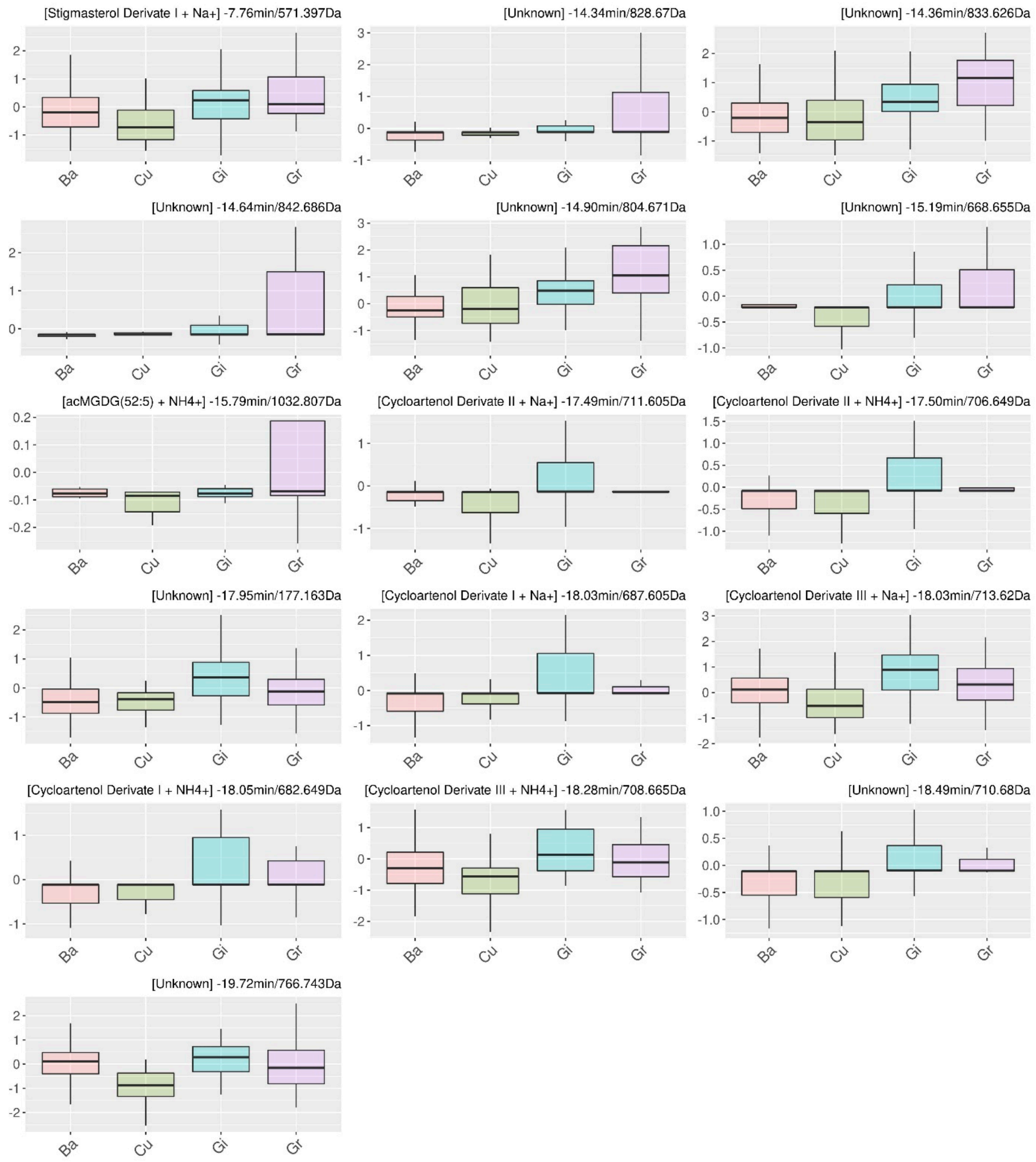

Figure S10. Boxplots of the autoscaled intensities for the features of the cluster A from the relation analysis for the determination of botanical variety in Figure 3b. Detailed information about the metabolites can be obtained from Table S3.

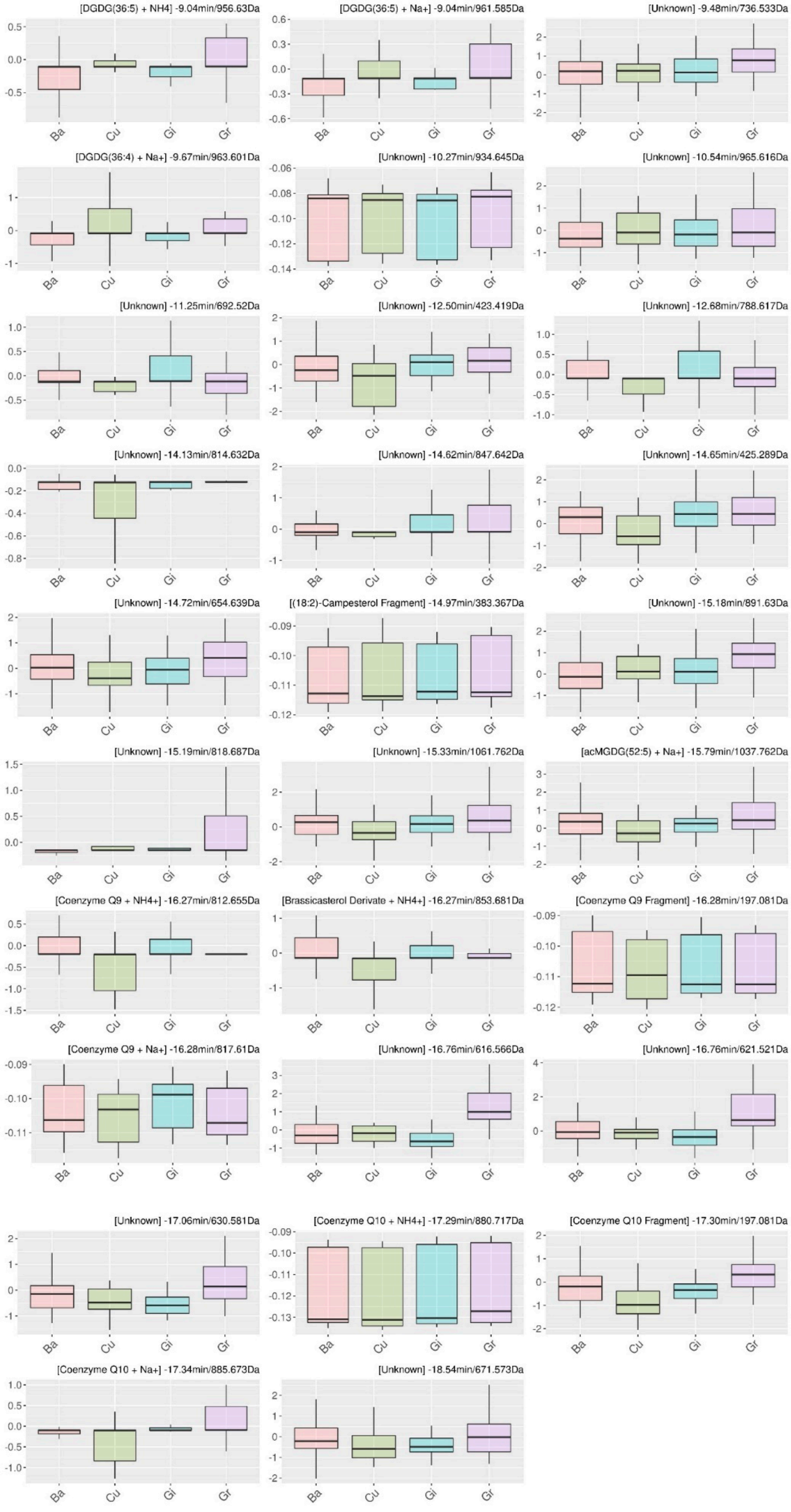

Figure S11. Boxplots of the autoscaled intensities for the features of the cluster B from the relation analysis for the determination of botanical variety in Figure 3b. Detailed information about the metabolites can be obtained from Table S3.

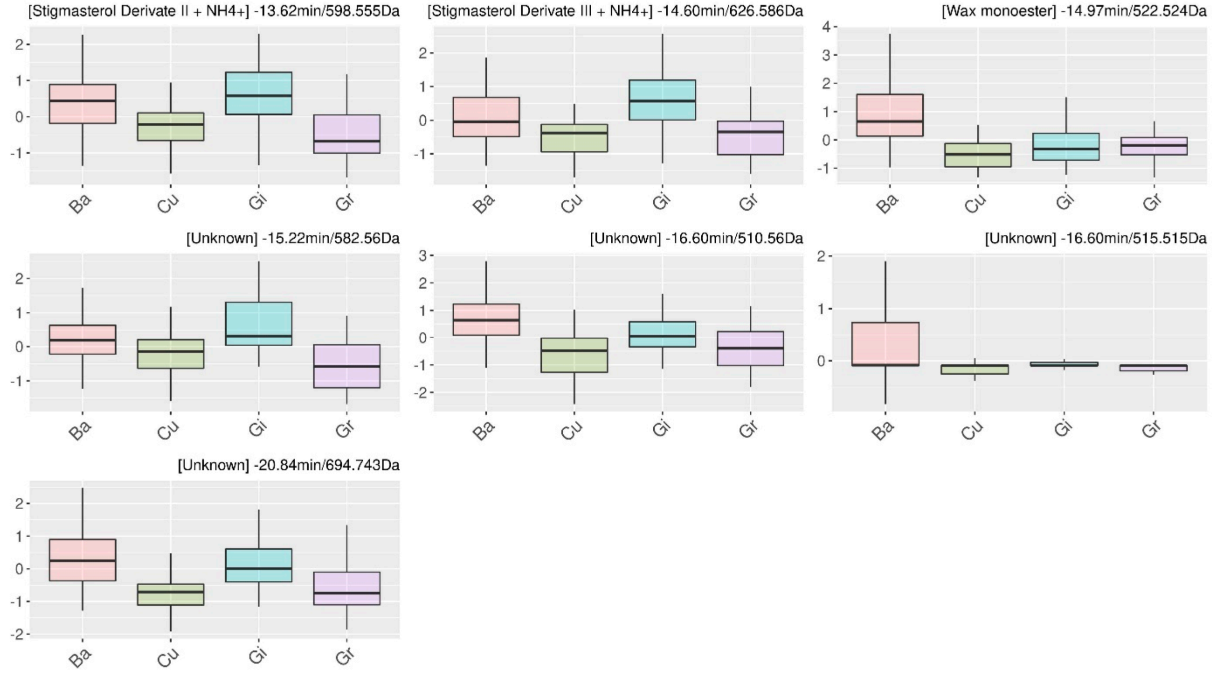

Figure S12. Boxplots of the autoscaled intensities for the features of the cluster C from the relation analysis for the determination of botanical variety in Figure 3b. Detailed information about the metabolites can be obtained from Table S3.

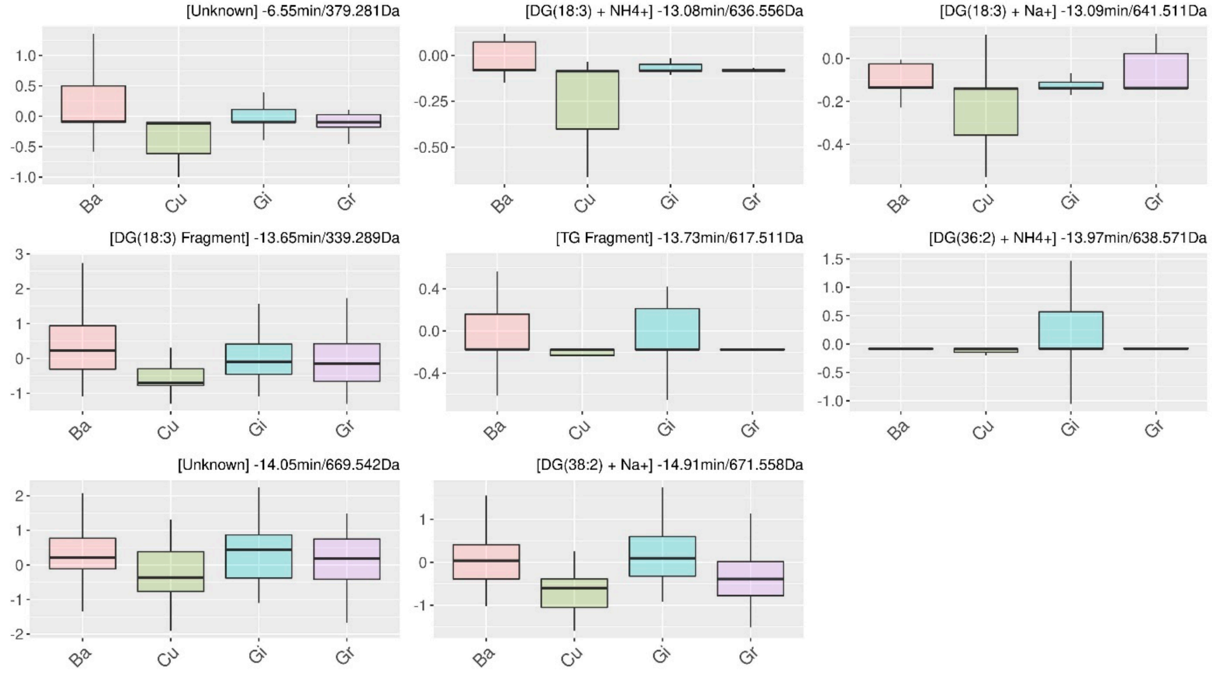

Figure S13. Boxplots of the autoscaled intensities for the features of the cluster D from the relation analysis for the determination of botanical variety in Figure 3b. Detailed information about the metabolites can be obtained from Table S3.

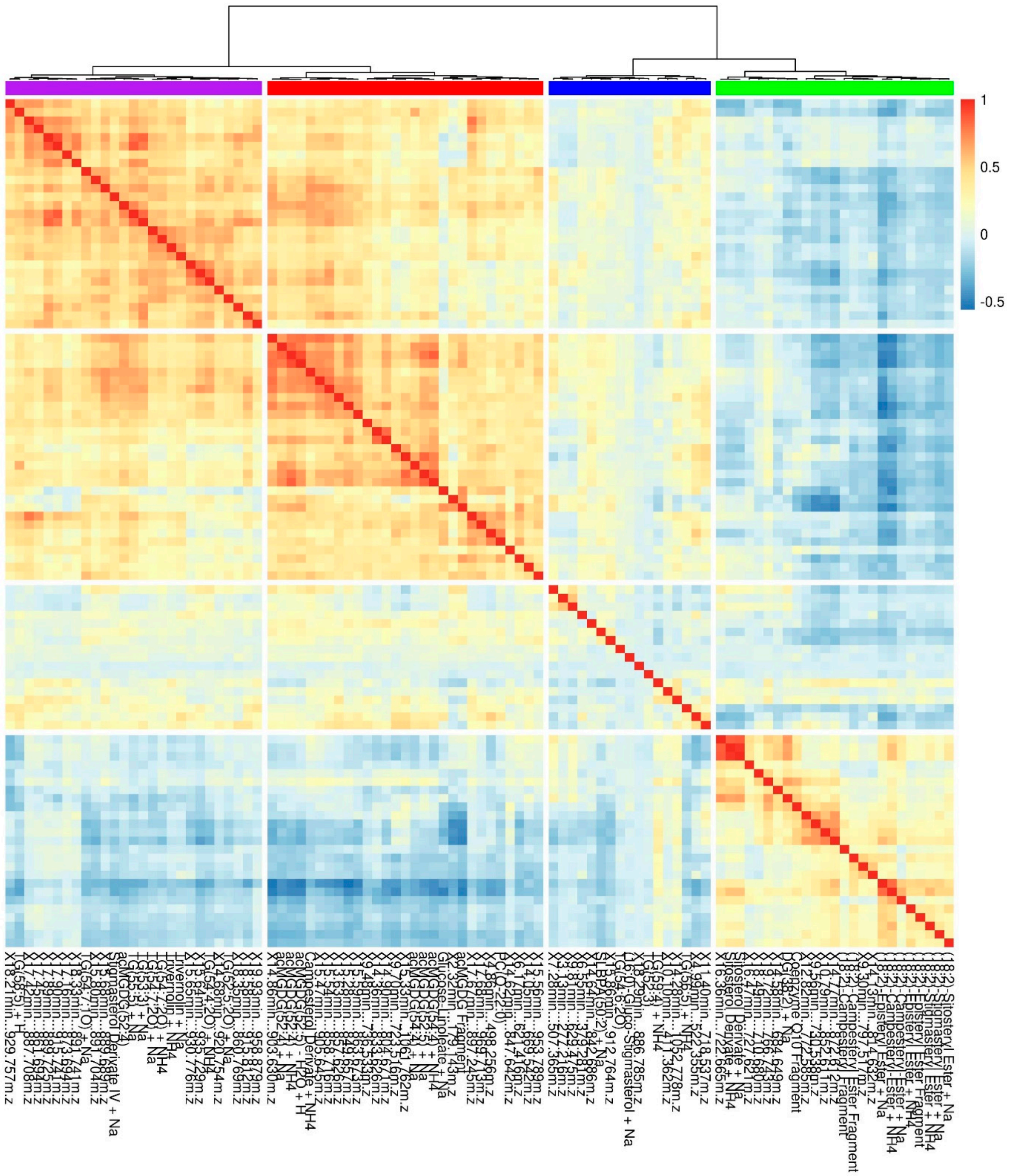

Figure S14. Results of the correlation analysis of features that were selected by SMD for the determination of geographical origin. For the hierarchical cluster analyses Euclidean distances and Ward algorithm were applied.

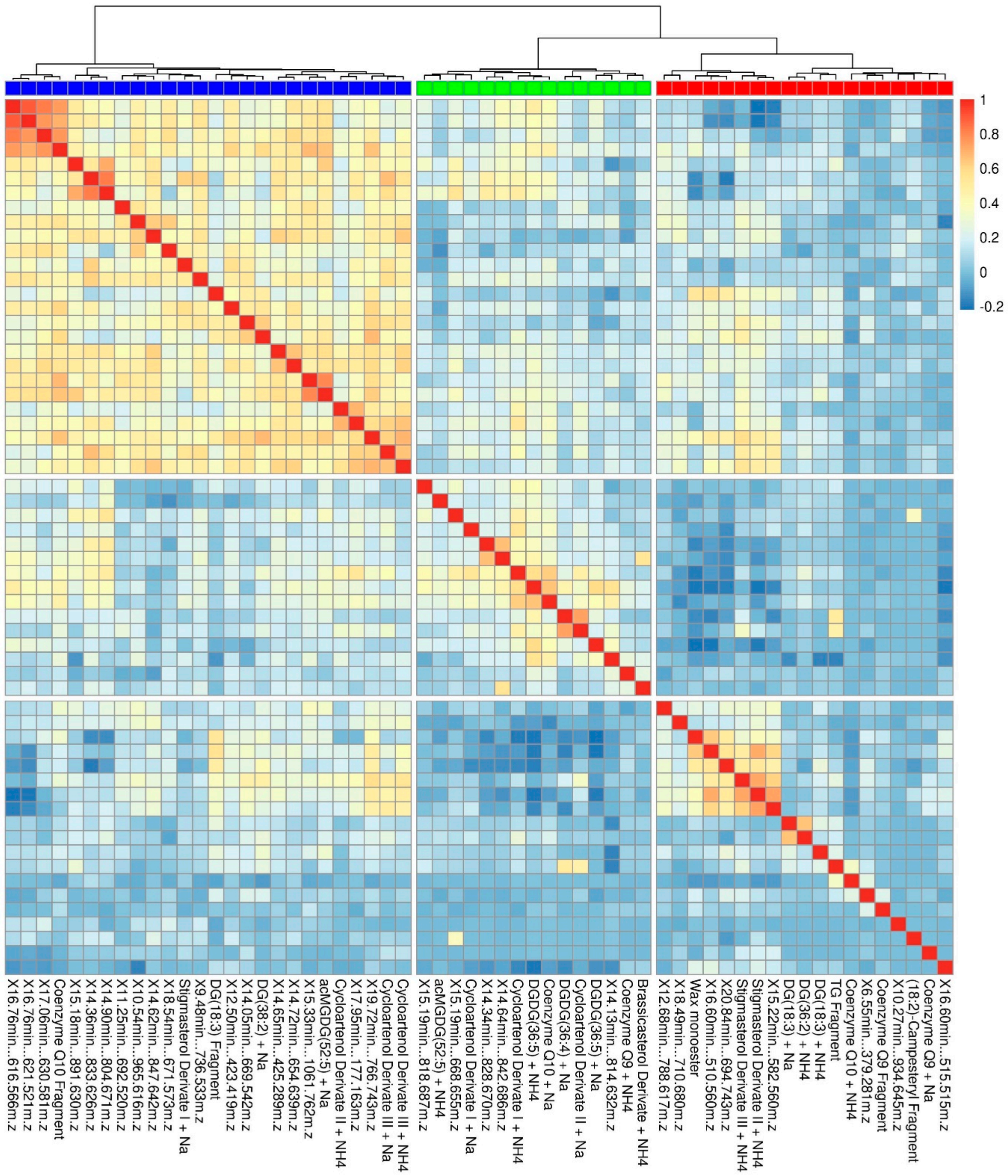

Figure S15. Results of the correlation analysis of features that were selected by SMD for the determination of botanical variety. For the hierarchical cluster analyses Euclidean distances and Ward algorithm were applied.

(a)

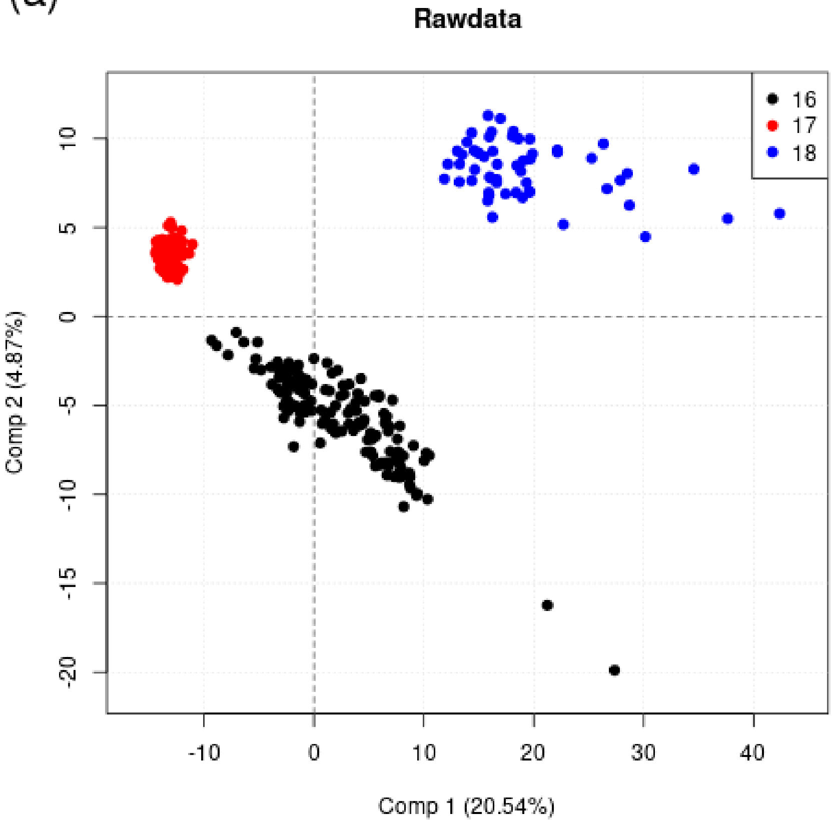

(b)

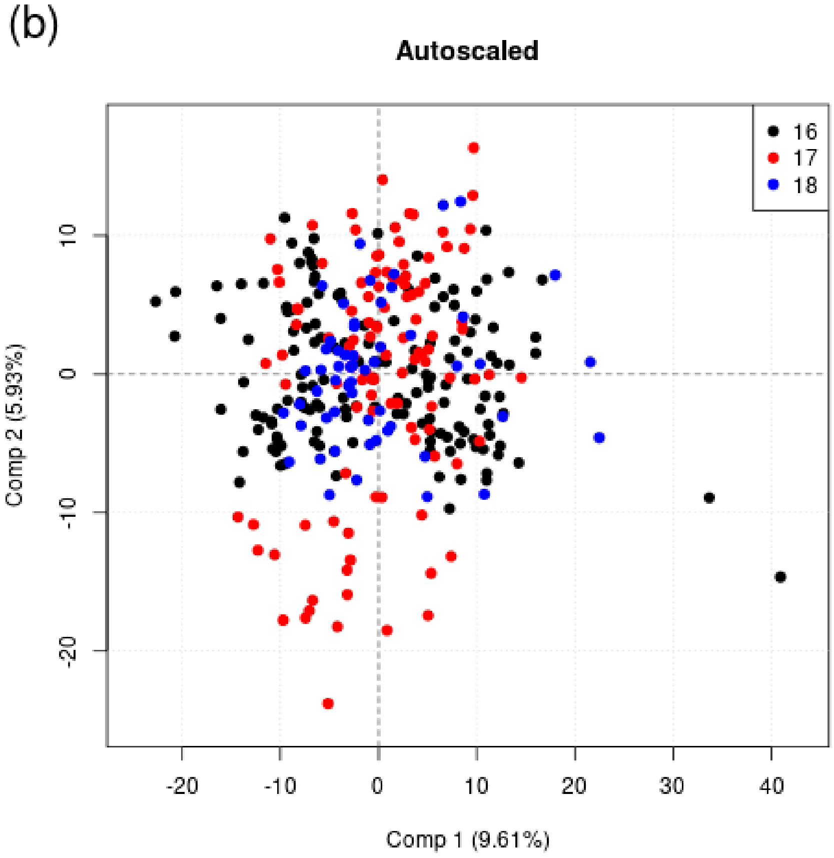

Figure S16. Scores of the first two principle components of the PCA applied to (a) raw and (b) autoscaled data. The different colors show the samples from the years 2016-2018 analyzed in three different batches.
