# Supplementary material for: Opening the Random Forest Black Box of the Metabolome by the Application of Surrogate Minimal Depth
Source: Metabolites. 2021 Dec 21;12(1):5. doi: 10.3390/metabo12010005 (PMC8781913; doi:10.3390/metabo12010005)
Supplement: Supplementary file 1 [file metabolites-12-00005-s001.zip › metabolites-1490989-supplementary/Supplementary Tables/TableS7_IdentifiedKeyMetabolites.pdf]

Table S7. Identified key metabolites of the asparagus samples with their LC-MS data, which show a dependency due to different origins.

Tentative compound	Proposed formula	Adduct	RT [min]	*m/z* measured	*m/z* calculated	Error [ppm]	Relevant fragments
**Glycolipids**							
Glucose-linoleate	$C_{24}H_{42}O_7$	$[M+Na]^+$	5.1	465.2818	465.2823	0.9	202.11; 145.05
**Glycerolipids**							
DG(36:2)	$C_{39}H_{72}O_5$	$[M+NH_4]^+$	14.0	638.5720	638.5718	-0.4	341.30; 337.27
DG(36:3)	$C_{39}H_{70}O_5$	$[M+NH_4]^+$	13.2	636.5566	636.5562	-0.7	339.29; 337.27
DGDG(36:4)	$C_{51}H_{88}O_{15}$	$[M+Na]^+$	9.7	963.5998	963.6015	-1.8	617.51; 599.50; 337.27
DGDG(36:5)	$C_{51}H_{86}O_{15}$	$[M+NH_4]^+$	9.1	956.6298	956.6305	-0.7	615.50; 597.49; 337.27; 335.26
TG(52:3)	$C_{55}H_{100}O_6$	$[M+Na]^+$	18.9	879.7421	879.7412	1.0	601.52; 577.52; 575.50
TG(52:5; 2O)	$C_{55}H_{96}O_8$	$[M+Na]^+$	15.1	907.6981	907.6997	1.8	597.49; 575.50; 574.49
TG(53:3)	$C_{56}H_{102}O_6$	$[M+Na]^+$	18.7	893.7562	893.7569	0.7	/
TG(54:4;2O)	$C_{57}H_{102}O_8$	$[M+NH_4]^+$	16.3	932.7909	932.7913	-0.4	617.54
TG(54:6;2O)	$C_{57}H_{98}O_8$	$[M+Na]^+$	15.1	933.7143	933.7154	1.2	617.51; 599.50; 337.27
TG(54:6;3O)	$C_{57}H_{98}O_9$	$[M+NH_4]^+$	14.1	944.7536	944.7520	1.4	613.48; 599.50
TG(54:7;1O)	$C_{57}H_{96}O_7$	$[M+Na]^+$	15.5	915.7037	915.7048	1.2	893.72; 613.48; 599.50
TG(54:7;2O)	$C_{57}H_{96}O_8$	$[M+NH_4]^+$	15.8	926.7442	926.7443	-0.1	611.47; 599.50
TG(55:5)	$C_{58}H_{102}O_6$	$[M+Na]^+$	18.3	917.7585	917.7569	-1.7	615.53; 599.50
TG(58:4)	$C_{61}H_{110}O_6$	$[M+NH_4]^+$	19.6	956.8651	956.8641	-1.1	659.60; 599.50
TG(58:5)	$C_{61}H_{102}O_6$	$[M+H]^+$	18.7	931.7767	931.7749	1.9	652.56; 629.55; 599.50
**Phospholipids**							
PC(16:0)	$C_{24}H_{50}NO_7P$	$[M+H]^+$	4.8	496.3407	496.3398	1.9	184.07
PC(O-22:0)	$C_{30}H_{62}NO_7P$	$[M+H]^+$	7.7	580.4336	580.4337	-0.1	184.07
**Phytosterols**							
Brassicasterol derivative	$C_{56}H_{85}NO_4$	$[M+NH_4]^+$	16.2	853.6807	853.6817	-1.2	381.30
Campesterol derivative	$C_{56}H_{100}O_7$	$[M+NH_4]^+$	17.6	902.7811	902.7807	-0.4	383.36
Cycloartenol derivative I	$C_{46}H_{80}O_2$	$[M+NH_4]^+$	18.3	682.6499	682.6497	-0.4	409.38; 191.18

Table S7– continued.

Cycloartenol derivative II	$C_{48}H_{80}O_2$	$[M+NH_4]^+$	17.8	706.6505	706.6497	-1.2	409.38; 191.18
Cycloartenol derivative III	$C_{48}H_{82}O_2$	$[M+NH_4]^+$	18.4	708.6652	708.6653	-0.1	409.38; 191.18
Sitosterol derivative	$C_{44}H_{82}O_5$	$[M+Na]^+$	16.0	713.6041	713.6054	-1.8	397.38
Stigmasterol derivative I	$C_{33}H_{56}O_6$	$[M+Na]^+$	7.8	571.3965	571.3969	-0.7	395.37
Stigmasterol derivative II	$C_{40}H_{68}O_2$	$[M+NH_4]^+$	13.6	598.5559	598.5558	0.2	395.37
Stigmasterol derivative III	$C_{42}H_{72}O_2$	$[M+NH_4]^+$	14.8	626.5864	626.5871	1.1	395.37
Stigmasterol derivative IV	$C_{57}H_{100}O_7$	$[M+Na]^+$	17.3	919.7356	919.7358	0.3	395.37
**Ubiquinones**							
Coenzyme Q9	$C_{54}H_{82}O_4$	$[M+NH_4]^+$	16.5	812.6558	812.6551	-0.8	197.08
Coenzyme Q10	$C_{59}H_{90}O_4$	$[M+NH_4]^+$	17.5	880.7191	880.7177	1.5	197.08
**Waxes**							
Wax monoester	$C_{34}H_{64}O_2$	$[M+NH_4]^+$	14.7	522.5240	522.5245	0.9	/

Abbreviations: DG, diacylglycerol; DGDG, digalactosyldiacylglycerol; PC, phosphatidylcholin; TG, triacylglycerol
